# Supplementary material for: Efficacy and Feasibility of the Minimal Therapist-Guided Four-Week Online Audio-Based Mindfulness Program ‘Mindful Senses’ for Burnout and Stress Reduction in Medical Personnel: A Randomized Controlled Trial
Source: Healthcare (Basel). 2022 Dec 14;10(12):2532. doi: 10.3390/healthcare10122532 (PMC9778772; doi:10.3390/healthcare10122532)
Supplement: Supplementary file 1 [file healthcare-10-02532-s001.zip › Audio scripts S1.pdf]

Appendix SA. Audio scripts of four mindfulness practice guided audios that were used in the MS program (translated from the original Thai version)

1st mindfulness audio - Stress relief by yourself

Please sit in a comfortable position. You may choose to close your eyes.
Then, gently explore each sensation that comes through your body at the moment.
How does it feel between the eyebrows? Tense or relax?
Just feel the sensation as it is—no need to try to relax.
Feel the sensation of blinking eyelids.
Sense the breath going through your nostrils and throat.
At the moment, is the breath warm, hot, or cool?
What odors can you smell right now?
Feel the movement of your chest and belly when you breathe in and out.
Feel the expansion when breathing in and deflation when breathing out.
Feel the touch of the clothes that contact your skin.
At the moment, how does your skin feel? Hot, cold, or cool?
Feel the sensations at both of your palms.
Feel the sensation at your body parts that are contacting the floor.
Feel the weight that pressures those body parts.
At the moment, are your arms and legs tense or relaxed?
What you just did is to feel your body sensations in each present moment.
This technique will make a space for your mind to rest when you are stressed.
Help you to pause from thinking of things that make you worried, irritated, or depressed,
and, instead, be with your body sensations in each moment.
You can use this technique anytime, anywhere, and as much as you like.
When you master it, the stress in your life will substantially decrease.
Explore sensations on each part of your body again.
At the moment, how does your skin feel? Hot, cold, or cool?
At the moment, are your arms and legs tense or relaxed?
----------- (pause) -----------
While you are feeling the sensations from each body part,
some thoughts may come to your mind.
No matter what those thoughts are, gently let them go.
Then, continue to feel the sensations through your body again.
Explore sensations on each part of your body again.
Sense the breath going through your nostrils and throat.
Feel the movement of your chest and belly when you breathe in and out.
----------- (pause) -----------
If some thoughts come into your mind again, just know that you are thinking.
Gently let those thoughts go.
Then, return to feel the sensations through your body in each moment again.
Feel the sensations at both of your palms.

Feel the sensation of blinking eyelids.
Feel the tension between the eyebrows.
----------- (pause) -----------
If some thoughts come into your mind again, gently let those thoughts go once more.
Then, continue to feel the sensations through your body again.
No need to blame yourself for thinking. It is human nature to think all the time.
We cannot prevent the arising of our thoughts.
However, we can train to be aware of our thoughts
and let them go when thinking is unnecessary
by directing your attention back to the feelings of your body at the moment,
like what you just did.
Every time you realize that you are overwhelmed with stress,
just let your thoughts go and feel your body.
When the negative thoughts are let go, the stress will also disappear.
----------- (pause) -----------
If you are thinking anything right now, gently let those thoughts go.
Then, feel the sensations at both of your palms again.
Sense the breath going through your nostrils and throat.
Feel the movement of your chest and belly when you breathe.
So many times that we cannot change the things around us.
However, we can always take our minds to rest.
Stress can be relieved every time you return to feel your body.

2nd mindfulness audio – Thoughts awareness, and letting go of thoughts

Please sit in a comfortable position. You may choose to close your eyes.
Listen to the sounds around you at this moment.
What sounds do you hear?
For each second passing by, each sound around you gradually changes.
Listen to these surrounding sounds for a moment.
Listen like listening to music—no need to think of anything.
----------- (pause) -----------
Each sound that you heard seconds ago was called outer sound.
Now, please slowly count the numbers 1-10 in your mind.
Can you hear the voice counting numbers in your mind?
This is called the inner voice.
Or our thoughts.
----------- (pause) -----------
Right now, the inner voices may be saying something.
----------- (pause) -----------
They may be asking questions.
----------- (pause) -----------
The inner voices talk all day.
We may sometimes notice them, and sometimes not.
----------- (pause) -----------
At the moment, the inner voices may be saying something.
----------- (pause) -----------
Now, listen to the outer sounds again.
What sounds do you hear?
Just listen continually—no need to think of anything.
----------- (pause) -----------
You may notice that when you listen to the outer sounds,
the inner voices may periodically interrupt.
----------- (pause) -----------
Your attention may sometimes focus on your thoughts
and make you stop listening to the outer sounds.
----------- (pause) -----------
At the moment, if some thoughts come into your mind,
gently let those thoughts go.
Then, continue listening to the outer sounds again.
----------- (pause) -----------
If inner voices continue speaking,
just know that you are thinking.
Let those thoughts go.
Then return to listen to the surrounding sounds again.
----------- (pause) -----------

If any thoughts arise, let them go.
Then, continue listening to the outer sounds once more.
----------- (pause) -----------
You may notice your thoughts more obviously
when the inner voices say something
while you are listening to the surrounding sounds.
----------- (pause) -----------
What you just did is the training of thought awareness and letting go.
Training to discriminate between outer sounds and inner voices
will help you to be aware of your thoughts more often
and let them go easier.
----------- (pause) -----------
Thoughts may start speaking again.
Gently let them go and return to listen to the outer sounds again.
----------- (pause) -----------
Every time you suffer from repetitive thinking,
just pause yourself from listening to your inner voices
and listen to the surrounding sounds continually.
Suffering will gradually subside.
Many times we try to think of the solution to unsolvable problems
or problems that cannot be solved at the time,
and suffer from thinking about it again and again.
Stop thinking, be with the present moment by listening to the outer sounds continually
can also make your suffering go away.
----------- (pause) -----------
Now, if any thoughts come in your mind,
stop thinking those thoughts.
Then, return to listen to the outer sounds as before.
----------- (pause) -----------
If the inner voices begin to speak again,
just know that you are thinking.
Let the thoughts go and continue listening to the surrounding sounds again.
----------- (pause) -----------
You can train to discriminate between outer sounds and inner voices like this all day.
Either when you are taking a shower, brushing your teeth, driving a car, waiting for a bus, or any other activities.
When you are more skillful,
you will be able to recognize more often the thoughts that make you suffer
and let them go faster, not keep thinking about them for a long time.

3rd mindfulness audio - Dealing with negative feelings

Please sit in a comfortable position. Do not close your eyes.
Then, gently look around you at the moment.
What do you see?
----------- (pause) -----------
The image that you are seeing
is the image of the situation that is happening at this moment.
Then, without closing your eyes,
try to think of what you have done today since you woke up.
----------- (pause) -----------
You may notice that
when you were thinking
the image of your surroundings would be faded
and your mind would, instead, focus on the mental images.
The mental images are the appearances of thoughts.
When we are thinking,
inner voices and mental images will appear,
sometimes obviously, sometimes not.
----------- (pause) -----------
Now, if any thoughts arise,
gently let them go.
and continue looking at the things around you.
Look at the things that are occurring in the present moment.
----------- (pause) -----------
If your mind return to focus on the mental images,
just aware that you are thinking again.
Gently let those thoughts go.
Then, return to look at the things around you as before.
----------- (pause) -----------
Mental images that arise are often about the past or the future.
----------- (pause) -----------
At the moment, if any thoughts arise,
gently let them go.
Then, return to look at the things around you as before.
Just look at them—no need to think of anything.
----------- (pause) -----------
When our minds focus on mental images,
our feelings will change along with those images.
We sometimes feel terrible for a long time with some mental images that keep coming up
Gently let those memories or imaginations go.
Come back to be with real frontal images and look at things around us.
Just look at them—no need to think of anything.

----------- (pause) -----------
Now, if some thoughts come into your mind,
gently let those thoughts go.
Then, continue to look at the things around you as before.
----------- (pause) -----------
You may gradually sense the lightness in your mind
that is different between when you are with those images that your mind created
and when you just be with the present images.
----------- (pause) -----------
While you are looking at the things around you,
inner voices may start speaking again.
Mental images may periodically arise.
Gently let those thoughts go.
Continue to look at the things around you.
Just look at them—no need to think of anything.
----------- (pause) -----------
There are several types of negative feelings, for example,
irritated, annoyed, sad, guilty, tense, worried, bored, hopeless.
When we are having these negative feelings,
there will always be some mental images that come through our minds.
They may be images of the past events or images that our minds created.
The more we take time focusing on these mental images,
the more negative feelings will be amplified and stay in our minds for a long time.
We can deal with these negative feelings by
letting go of those mental images.
Bring our mind back to focus on the surroundings.
Continually look at the things around us.
Just look at them—no need to think of anything.
For a while, those negative feelings will gradually disappear.
Please look at the things around you as before.
----------- (pause) -----------
Right now, if some thoughts come into your mind,
gently let them go.
Then, continue looking at the things around you once again.
----------- (pause) -----------
If inner voices begin to speak or mental images appear again,
gently let those thoughts go.
Then, return to look at the things around you as before.
----------- (pause) -----------
Some life event images may repeatedly play in your mind.
This is the nature of our mind.
We cannot prevent our thoughts to arise.
However, we can train to let them go anytime we are aware that we are thinking.

When our mind focuses less on those mental images,
they will gradually have less influence over our feelings.
Please look at the things around you again.
----------- (pause) -----------
At the moment, if any thoughts arise,
gently let them go,
and return to look at the things around you as before.
----------- (pause) -----------
If inner voices begin to speak or mental images appear again,
gently let those thoughts go.
Then, continue to look at the things around you again.
----------- (pause) -----------
At the moment, if any thoughts arise,
gently let them go,
and return to look at the things around you as before.
----------- (pause) -----------
You can bring your mind back to be in the present moment like this at anytime
by looking at the things around you instead of focusing on your mental images.
Stop repeatedly thinking about your past events or imagination.
After training for some time,
you will gradually be better at dealing with the negative feelings.
Your sufferings will be subsided and short-lived

4th mindfulness audio - Being in the present moment and letting go

Please sit in a comfortable position. Do not close your eyes.
Now, feel the sensations at the palms of both of your hands.
Feel the breath moving in and out from your nostrils and throat.
Feel the movement of your chest and belly when you breathe in and out.
Feel the sensations at the soles of both of your feet.
Listen to the surrounding sounds at the moment.
Just listen continually, like listening to music.
No need to think of anything.
Then, look at the surroundings.
Look continually at the things around you.
Just look—no need to think of anything.
----------- (pause) -----------
What you just did is called being in the present moment.
Feeling the sensations occurring throughout your body at the moment.
Listening to the surrounding sounds at the time.
Looking at the things happening in front of you.
Please be in the present moment like this for a while.
----------- (pause) -----------
If any thoughts arise,
no matter they are either inner voices or mental images,
let those thoughts go,
and bring your mind back to be in the present moment like before.
----------- (pause) -----------
Feel the tension between the eyebrows.
Feel the movement of your chest when breathing in and out.
Listen to the sounds around you.
Look at the things around you.
Feel the sensations at the palms of both of your hands.
----------- (pause) -----------
If any thoughts arise,
let those thoughts go,
and return to be in the present moment again.
----------- (pause) -----------
Look at the things around you.
Feel the breath moving in and out from your nostrils and throat.
Listen to the surrounding sounds.
----------- (pause) -----------
During this time, the inner voices may start speaking again.
Let those thoughts go
and continue to be with the present moment.
Feel the sensations throughout your body, listen to the surrounding sounds,

and look at the things around you.
----------- (pause) -----------
Negative feelings will occur when our mind focus on negative thoughts.
When we bring our mind back to be in the present moment,
those negative feelings will gradually disappear.
It will always be like this.
----------- (pause) -----------
Right now, if any thoughts arise,
let them go,
and come back to be in the present moment again.
Listen to the surrounding sounds at the moment.
Continually look at the things around you.
Just look—no need to think of anything.
Feel the breath moving in and out from your nostrils and throat.
----------- (pause) -----------
If the inner voices begin to speak again,
let those thoughts go,
and come back to be in the present moment again.
Feel the sensations at the palms of both of your hands.
Feel the sensations at the soles of both of your feet.
----------- (pause) -----------
After you practice mindfulness for a while,
you may have seen that
the negative thoughts and feelings will periodically arise in everyday life.
We cannot prevent these thoughts or feelings from happening.
The more we try not to think or feel, the more we suffer.
At this point, the only thing we can do is to let go,
understand, and accept the nature of being human.
What we can do is to be aware that we are focusing on negative thoughts
and making negative feelings grow.
Then, let go of those negative thoughts.
Bring your mind back to be in the present moment.
When you can let go of the negative thoughts, the negative feelings will also disappear.
----------- (pause) -----------
At the moment, if any thoughts arise,
gently let them go,
and bring your mind back to be in the present moment.
Feel the breath moving in and out from your nostrils and throat.
Continually look at the things around you.
Feel the sensations at the soles of both of your feet.
----------- (pause) -----------
Mindfulness practice is the practice of letting go.
Letting go of the things that we cannot control.

We cannot prevent our thoughts and feelings from arising,
but we can be aware of them and let them go anytime they occur.
----------- (pause) -----------
If some thoughts come into your mind,
gently let them go,
and bring your mind back to be in the present moment again.
Feel the tension between the eyebrows.
Feel the movement of your belly when you breathe in and out.
----------- (pause) -----------
Right now, if any thoughts arise,
let them go,
and bring your mind back to be in the present moment like before.
----------- (pause) -----------
If the inner voices begin speaking again,
let those thoughts go,
and be in the present moment once again.
----------- (pause) -----------
Every thought comes and goes.
Every feeling comes and goes.
No matter how huge the happiness or suffering,
they will also come and go.
----------- (pause) -----------
If any thoughts arise right now,
gently let them go.
Then, come back to be in the present moment again.
Look at the things around you.
Just look—no need to think of anything.
Continually listen to the surrounding sounds.
----------- (pause) -----------
If the inner voices start speaking again,
gently let those thoughts go,
and continue listening to the surrounding sounds as before.
----------- (pause) -----------
Keep being in the present moment like this for the whole day.
When your mind becomes more familiar with being in the present moment
rather than wandering in the thoughts,
suffering in your life will substantially subside.
Use your thoughts only when you need to work or think.
Let your mind rest when thinking is unnecessary.
Bring your mind back to its home.
Being in the present moment.
