# Supplementary material for: Efficacy and Feasibility of the Minimal Therapist-Guided Four-Week Online Audio-Based Mindfulness Program ‘Mindful Senses’ for Burnout and Stress Reduction in Medical Personnel: A Randomized Controlled Trial
Source: Healthcare (Basel). 2022 Dec 14;10(12):2532. doi: 10.3390/healthcare10122532 (PMC9778772; doi:10.3390/healthcare10122532)
Supplement: Supplementary file 1 [file healthcare-10-02532-s001.zip › Daily messages S1.pdf]

Appendix SC. Contents in the daily messages regarding practical points in mindfulness practice used in the MS program

Day 1

In mindfulness practice, we keep paying attention to an object and being aware when attention is off focus and be with thoughts. When attention is drawn to thoughts, gently direct attention back to the object. If mind wanders and attention is drawn to thought again, gently direct attention back to the object again. Repeat the practice process like this over and over. You can call the object that you keep paying attention while practicing mindfulness as "home" of mind. (It is sometimes known as base of mind).

In this 1st audio file, we will start practicing mindfulness by using body sensation as the home of mind. The goal is not trying to achieve the state of no thinking, but to see that we actually think of many things in a day.

Mindfulness practice helps you be more present and suffer less. Mindfulness is a skill that can be practiced like other skills. The more you listen to the audio file, the more you are aware of things in your mind.

Day 2

Some people may start to notice that our mind does not stay still. It thinks about something all the time. This is the nature of our minds. Be aware when your attention is not at body sensation, then gently bring it back to body sensation over and over again.

When attention return to home (body sensation), you may notice that your mind can take a break from overwhelming thoughts.

One good remedy for burnout is to rest. When you take a break, the fire within you could be gradually reignited again. Bringing attention back to body sensation is a kind of mental rest that we can do as often as we want in a day. We can do it anytime and anywhere. Taking a break from stressful thoughts for a while, maybe just 10 minutes, is enough to feel refreshed.

In a day, we may have lots of tiring things to think of. Knowing how to rest the mind, like what you are practicing, can help relieve your tiredness each day.

Day 3

Once you get used to this method of mindfulness practice, please try to apply the practice in the 1st audio file in your daily life. For example, when taking a shower, you can pay attention to the sensation on your body where water flows through, perceive the warmth or coldness of the water, recognize the smell of shampoo or soap, feel the smoothness and bubbles of soap while rubbing your body. In the meantime, if your mind wanders, put down those thoughts and gently direct your attention back to body sensations. When you walk to work, you may pay attention to the feeling at both soles in each step, feel the warmth and coldness you perceived on the skin, feel the wind hitting your body or face. If you are driving or sitting in a car to work, you may feel the softness of the seat touching your back, feel the pressure of your buttock against the seat, feel the sensation of your hands holding the steering wheel. You may apply the mindfulness practice when working or doing housework as well. Applying the practice in daily life will make your mindfulness skills develop fast, and you will experience the change in yourself within a short time. Let's try it out!

Day 4

Some people have sleep problems such as insomnia, waking up in the middle of the night, and having difficulty falling asleep. You can apply the techniques in the 1st audio file to help you sleep easier.

We usually sleep when our mind is calm and our body is relaxed. We may sometimes be unable to sleep because we keep thinking about something such as work, worrying about what will happen or need to be done tomorrow, thinking about the grouchy things in the day, etc. You can try to bring your attention to body sensation instead of those thoughts. If some thoughts pop up in your mind, you may pause yourself from being caught up in those thoughts and return to feel the sensations in the body as in the 1st audio file. This technique will calm your mind down and you will be able to sleep easier. If you wake up in the middle of the night and have difficulty falling asleep, you can use this technique as well.

If there is something that you need to think about and you are afraid that you will forget it, you may write down the things that you need to consider on a piece of paper and put that paper next to your bed. Then, you can stop thinking for tonight and start thinking about it again next morning.

Day 5

Some of you may have practiced other mindfulness techniques before, such as practicing mindfulness by focusing on breath or expansion and contraction of stomach. These kinds of practice are considered as paying attention to body sensation like in the 1st audio file as well. However, mindfulness techniques that use breath or expansion and contraction of stomach as an object of attention allow your attention to focus on only one part of your body, not paying attention to many parts of body like the technique we used in the 1st audio file.

Being able to focus on many parts of the body will make it easier to apply the practice in daily life. For example, when we wash dishes, we can recognize the feeling of the hands touching some water, feel the weight on the soles of our feet, feel the tension in the neck when we bend down.

When practicing mindfulness, you may not always be calm. Some days you may be very distracted. It is okay to think about many things in a day. This is the nature of mind. If you can realize that the mind is often wandering, it means that your mindfulness skill is improved. Because from the beginning, we are thinking all day with very little awareness of our thinking, and then we realize how often we are thinking in a day.

Day 6

This 2nd audio file will use the surrounding sounds as the home of mind instead of body sensation. This audio file will help you see your thoughts more clearly and often. Thoughts often come in the form of inner voices. When you can distinguish between external and internal sounds, you will hear your thoughts more often.

Why does mindfulness practice focus on knowing the thought? It is because the main cause of human suffering is thoughts. In the same situation, if you think differently, you will feel differently. For example, your friend walked passing you without saying hello. If you think he might not see you, you may feel nothing. On the other hand, if you think that he might be angry with you, you will be worried.

Some people may practice seeing things from different perspectives to help them feel better in many situations, which is a good strategy. However, when you cannot see a bad situation in other ways, you may be overwhelmed with negative thoughts. The longer you sink in those thoughts, the more and the longer you feel bad. Awareness that you are thinking and this is just a thought will help you in this situation. You may realize that you suffer because you are caught up in those negative thoughts. Then, you can choose whether you will continue to be drained into those negative thoughts or return your attention to its home by focusing on your body sensation or surrounding sounds.

For this reason, being aware of one's thoughts is the first and most essential step in dealing with our emotions.

Day 7

During the day, please try to keep listening to the sounds around you. You will find that there are many sounds that you have rarely or never heard of before. If you keep listening to the sounds, you will find that you can hear the voices in your mind clearer and more often as well.

External sounds and internal voices have the same characteristics. They will change from time to time. When one voice or sound ends, another will come to replace it. Some voices in our mind that we are obsessed with can make us unconsciously tired. It can also be another cause of burnout. A stressful job, heavy workload, or problem with coworkers can trigger some complaints in your mind. However, these difficulties do not make each person tired equally. What makes our level of tiredness different?

People who feel more tired are the people who cannot stop paying attention to their inner voices. Even though a frustrating event was over some minutes ago, the complaining voices in their mind still remain. They constantly pay attention to their complaining inner voices even after work, at home, while eating, taking a shower, sitting, lying down, or when waking up the next morning. This could make them really mentally fatigued. When the mind works all the time and never rest like this, it would typically be distressed, and the life energy would gradually run out.

On the other hand, people who can rest their minds and let go of the troubling things that happened. While taking a shower, they feel the water flowing through the body. When they sit and rest, they feel the softness of the chair. When they lie down, they listen to the sound of an air conditioner or a fan. If the mind can rest at the proper time, it is like charging a battery of life. You will be ready to deal with new problems in the next day.

During the day, if we can bring our attention to listen to the surrounding sounds continuously, we will be able to rest and recharge as often as we want.

Day 8

Surrounding sounds will change continuously throughout the day. Some sounds are loud and some are low. Some sounds are beginning and some are fading away. When you wake up in the morning, there may be sound of wind from an air conditioner or a fan, sound of silence (the "Chi" sound in the air when the room is quiet), or sound of a clock ticking. When you brush your teeth, there is sound of a toothbrush brushing your teeth, sound of water flowing from a faucet. While taking a shower, there is sound of water dropping on the body or the floor. When you get dressed, there is sound of clothes rubbing against skin. When you eat, you can hear sound of rice chewing, sound of a fork hitting a plate. When you drink, you can hear sound of water swallowing. During the day, there is sound of people talking, TV, music, birds chirping, footsteps, car engine, etc.

You may just listen to surrounding sounds without need for thinking anything. Let each sound come and go. Keep listening like listening to music. It could help you calm your mind. Just listen to those sounds whether you like them or not. When you can let those sounds come and go, you will be less distressed. We cannot change many things around us.

When we get used to non-selectively listening to surrounding sounds, we may understand that they are just sounds that will come and go. Try listening to your mental voices with this attitude as well.

Voices in your mind may sometimes complain about someone, worry about the future, or blame yourself. Whether you like those mental voices or not, just let them come and go like those surrounding sounds. You do not need to correct your thoughts. Then, you may find peace in your mind when you can let mental voices come and go.

Day 9

How often do you notice your thoughts so far? What are thoughts that you frequently think about? Each person may have a different pattern and way of thinking. Some people might get angry easily, often complain about things or other people. They probably often think “Why does it go like this?”. Some people might not be confident in themselves. They probably often think “Will I be able to do it?”, “I am not smart”, “Will they hate me?”. Some people might be so strict with themselves. They probably often think “I should be more diligent”, “I should try harder”, “Why am I so lazy?”.

These thoughts can cause you latent stress. If you are caught up in these thoughts while doing activities, you could feel stressed even if activities that you are doing are not stressful like sitting, lying down, taking a shower, eating, driving, etc. You suffer because of thoughts, not what actually happens in the present moment.

Why do we have this kind of thinking pattern or mindset? Each thought that goes through our heads comes from life experiences and learning from parents, teachers, peers, books, media, etc. We will not deal with these stressful thoughts by trying not to think them. It is impossible to prevent thoughts from arising. They come up very quickly. If you force yourself not to think, you will suffer more when thoughts arise again. What we will do with these thoughts is to just watch, observe them, and accept that this is our thought pattern. Whether we like them or not, they have their causes and we cannot stop those thoughts from coming. You should realize that they are just thoughts, not facts. They are just points of view. The more you dive deep into these thoughts, the more you suffer. You can deal with these stressful thoughts by bringing yourself out of these thoughts. First, pause yourself from further thinking or drowning in these thoughts. Then, gently bring your attention back to surrounding sounds or body sensations.

If you are less caught up in thoughts and believe in them less, your unnecessary stress from thoughts will gradually decrease.

Day 10

Some thought patterns may unconsciously cause stress or burnout. For example, some people think about work all the time. They do not want to waste their time. They expect their work to be done quickly, so they think about work while eating, showering, driving, traveling, working out, or even hanging out with their friends. They do not allow themselves to rest until their works are done. On the positive side, it might help their works finished faster. If the amount of work is not much, they will be able to finish the work first and then take a break as they planned. However, in reality, our work is never really finished. When one job is finished, another comes in. There are

sometimes 2-3 more jobs coming in, even though the first job is not finished yet. In addition, many life challenges come at the same time such as money issues, family issues, and health problems. When we spend all of our time thinking about those problems, there is really no time left for us to rest as we originally intended.

The burden from overloaded work may be alleviated by reordering our priorities. The work does not always have to be completed before taking a break. Many jobs have their certain deadlines. We can prioritize the urgency of the job. Resting time is essential, so we need to focus on it as well. You can rest your body and mind while you practice mindfulness. Any work that is not urgent, you can gradually do it. You do not have to finish it today. Resting and taking care of your mind is not less important than working. Sometimes you might create your own deadlines that are too tight. For example, you want to complete work in 2-3 days, even though the actual deadline is one month.

Some people will be worried and cannot rest their minds if the jobs have not finished. The mindfulness practice can help with this problem. We may be overwhelmed with worry, although we are taking a break (like sitting or lying down comfortably). This happens because we are not in the present moment. You might learn from mindfulness practice that you are suffering because you keep paying attention to thoughts about work when you are taking a break. When you return your focus of attention to surrounding sounds or feelings occurring in your body, sufferings from thoughts will go away. You may notice that when you are focusing your attention on watching series, reading books, you are rarely worried about unfinished work. We suffer only when we are caught up in thoughts about unfinished work. If it is not a rush job, you should try to allow yourself to be rest and acknowledge the happiness in the present moment. You will have enough energy to continue your work the next day.

We may not suffer because of unfinished work, but we suffer because we are drowning in the fear that the work will not be finished.

Day 11

Our thoughts not only appear as the mental voices but also as mental images. They are sometimes pictures of what happened in the past, what we plan to do, and what we expect to happen. The 3rd audio file will help you to be more aware of thoughts that come in the form of images in your minds.

You may notice that you often focus on mental images rather than actual images in front of you. It seems that many times we are looking at the things in front of us. However, there are actually a lot of images in our minds, and we do not even know that we are paying attention to those images. For instance, when you are brushing your teeth in the morning, what you can see in front of you is a reflection of yourself in the mirror or a wash basin. However, your attention might not be with these images for long. Your attention might be with front images only for a short period, and then your attention is probably caught up with the images of yesterday's event, things that make you feel sad or happy, things you have to do today, things that you expect to happen, etc.

Some mental images might cause you strong emotions. Happiness and sorrow could come and go many times while we are brushing teeth. Let's look at how often your mind focuses on mental images in a day. Do you feel different between when you are paying attention to mental images and when you are paying attention to actual front images in the present moment?

Day 12

Thoughts can induce feelings and the feeling itself will induce the next thought. For example, at some point, we may be thinking about a colleague who exploited us. Then, we feel irritable. The irritable feeling might induce next frustrated thoughts such as thinking about when other people say bad things to you, a childhood friend who maltreated you. Then, the frustration in your mind grew even more. Thoughts and feelings can induce each other reciprocally. If we did not think about this exploitation, we would not get frustrated. Then, we would not be upset because we did not think about all of the other things.

This cycle of thought and emotion is called rumination. It is also sometimes called immersion into thoughts, or sinking into emotions. Being caught up in emotions or thoughts is the main cause of people's suffering. Real suffering is short-lived and comes from time to time, but the suffering caused by rumination can make us suffer more than we should, and suffering can be prolonged as long as we sink into rumination.

Rumination can happen to anyone but it is more common in people with depression. Depressed patients have long and frequent ruminations. They are often immersed in sad feelings or thoughts. Rumination is reduced in those who practice mindfulness. They can be aware of their rumination, and be able to choose to ignore those thoughts or emotions.

When we are more aware of the present moment by observing what is happening in front of us in everything we do, it will help us to be easily aware when we sink into ruminations and get out of the whirlpool of thoughts and emotions. As a result, our suffering will be reduced.

Day 13

As gas is the fuel of fire, thoughts are the fuel of emotion. When we turn on gas to cook, the fire can still be on. However, when we turn off the gas, the fire will go out. Likewise, we will only suffer when we pay attention to suffering thoughts. However, when we do not pay attention to those suffering thoughts, our suffering will fade away.

You may notice that you feel suffered when you repeatedly think about some suffering thoughts such as the day you fought with someone, what you think you should not have done, annoying behavior of people around you, etc. You will suffer only when you are paying attention to these thoughts. When you concentrate on something else such as watching a fun TV series or focusing on images in front of you, you are not suffered from these thoughts. When you are not focusing on anything, your mind will start wandering and keep thinking about miserable or annoying things, and that will make you suffer again.

The suffering you are experiencing might not be because of a fight or sad things that are happening right now. Instead, it is caused by your paying attention to the mental images of suffering events which makes them keep replaying over and over in your mind. It is like taking an arrow you have been shot once to stab yourself again at the same wound for tens or hundreds times with your own hand. We may not be able to correct what made us suffer in the past, but keeping thinking about it and making ourselves repeatedly suffer is unnecessary. Practicing mindfulness will help you to be more aware of repetitive negative thoughts.

Whenever you realize that you are caught up in suffering thoughts, gently return your attention to images in front of you. Keep doing like this over and over again. Your brain will gradually think less about those suffering thoughts. (The brain will think more of those thoughts if you keep paying attention to them. When you respond to those thoughts as merely thoughts that will come and go, and do not pay attention to them seriously, your brain will recognize those thoughts as unimportant and think less about them.)

Day 14

Have you ever had a thought pop up after you look at something and then continue thinking many thoughts for a long time before you realize that you are thinking? For example, when you are looking at the sky a thought like "today is very cloudy" might pop up in your head. Then, the train of thoughts continue like "will it rain at home or not?", then "yesterday, a colleague borrowed an umbrella and he has not returned it yet.", then "He borrowed my money and has not returned it too", and then "Another colleague also often refuses to work and takes advantage of me". As a result, you become distressed and bored to work. After a while, you might soon realize that you have been thinking about a lot of things and have been irritable and frustrated for some time just after only looking at the sky.

It is the nature of our mind that keeps us wandering through the flow of thoughts throughout the day. When we do not focus attention on front images, surrounding sounds, or body sensations, our attention will wander along the stream of thoughts, from one to another, and so on. Our mind often wanders to negative thoughts that make us feel annoyed, sad, disappointed, etc. Then, many thoughts in the same emotional theme will follow. It is like our mind is drained into a whirlpool. Sometimes we can come out, sometimes we cannot.

Mindfulness is like a bank for us to hold, preventing us from flowing along the stream of thoughts or being sucked into the vortex of emotions. Periodically directing our attention back to focus on the image in front of us will break the flow of thoughts and help us not sink into the emotional vortex.

If we can bring our attention to focus on the picture in front of us in every activity in a day, we will see the results of mindfulness practice faster and more clearly. We will sink less in emotions such as anger, sadness, fear, etc. When we close our eyes before sleeping, we can focus on the image in front of us as well. The front image that we can see when our eyes close is the darkness behind our eyelids with a slight light coming through our eyelids. If another image appears while our eyes are closing, that is your mental image or a thought. You may practice seeing mental images with your eyes closed as well. It can help you recognize your thoughts more often.

Day 15

How much we suffer depends on how much we believe in our suffering thoughts. For example, some people think and believe that they are incompetent, so they are not confident. They feel bad for themselves when they work with others because they believe that they will be burden for other people and others will see them as lazy colleagues, etc. Even though in reality, other people view them that they are really smart, kind-hearted, and generous, they are still suffered because they so believe in their suffering thoughts.

On the other hand, some people think and believe that they are more intelligent, smarter, and more experienced than others. They feel frustrated when other people disagree with their thoughts or refuse to do as they suggest. Actually, one thing can be viewed from many aspects. Each person has a different definition of what is good. Nevertheless, because they have already believed that their thoughts are correct, they feel distressed when they have to face different opinions.

Thought changes when we gain more experience. When we were young, we had one mindset. When we grew up, our mindset changed. We people think differently because each of us has different life experiences. Nevertheless, no one's thoughts are always 100% right because our experiences are limited. We are all like blind people who are touching an elephant, if we touch an

elephant's leg, we will think of elephants as pillars. If we touch an elephant's tail, we will think that the elephant is like a rope. If we touch the body, we will think that the elephant is like a wall.

Our thoughts are not entirely true. Thoughts are just some parts of the truth, and there are many parts of the truth that we cannot see. Letting go of your beliefs and knowing that they are just one perspective, not the whole truth. It will help you suffer less because of less attachment to thoughts. We will be able to view ourselves, others, and the world in a more balanced way. It is not all good and not all bad, but it can be viewed from many perspectives.

Day 16

We have gradually built homes for our mind called body sensation, surrounding sounds, and front images from practice following the past three mindfulness-guided audio files. In this 4th audio file, we will combine all homes of our minds into one big home called "the present moment".

In mindfulness practice, we practice by keeping paying attention to experiences in the present moment. It is the way to change the mental habit that always sinks in flow of thoughts. Every time you refocus your attention on the present moment (feel the body sensations, listen to the surrounding sounds and look at images in front of you), you are in the present moment.

In addition to body sensation, surrounding sounds, and front images, you can pay attention to other sensory modalities like smells or tastes you are perceiving as well.

When you are in the present moment and paying attention to experiences coming through your five senses, you will not suffer from unfinished work, irritating words you heard yesterday, your colleague's exploitation last week. You will not suffer from anything that is not happening right now.

The present moment is the home of your mind, that you can come back every time you are sad or tired from chasing after past or future thoughts. When you live in the present moment, suffering will be much less.

You can bring your attention to the present moment at any time. For example, when you eat breakfast, you may look at the rice on the plate, feel the touch of the hand holding the spoon, recognize the smell and taste of food, listen to yourself chewing food, listen to the sound of the fork hitting the plate, experience the warmth or coldness of the water we drink, feel the sensation in the throat when swallowing, etc. When you take a shower, you may look at the flowing water, listen to the sound of water hitting the ground, smell the smell of soap or shampoo, feel the slippery touch when rubbing soap on your skin, recognize the warmth or coldness of the water being touched, look at the shower, shampoo bottle, faucet, listen to the sound of the shampoo bottle opening, etc.

When your mind is in the present moment like this, not only your suffering will be reduced but you can also perceive small happiness that you normally do not perceive as well. For example, you may be able to sense pleasure from mild flavors hidden in food, many soft aromas hidden in each brand of coffee, calmness of mind when listening to the sound of running water or the sound of silence, feeling of relaxation when smelling shampoo or soap.

Let's try to notice how you feel when you live in the present moment.

Day 17

The mindfulness practice is neither trying not to think nor letting the mind wander through thoughts without realizing.

Mindful living or living in the present moment is something in the middle between the two extreme ways above. When you are in the middle way, your attention will alternate between being in present moment experiences and thoughts throughout the day.

If you try to focus on the experience too much, try not to think of anything, it could make you feel uncomfortable because you will not be able to do it and be frustrated. If you focus too much like this, you should let go more. You cannot stop thoughts from popping up. We can only be aware of them when they arise. After you notice your thoughts, you then let them go, so that you will not have to suffer for a long time with them.

If you muddle too much. Your attention is being with thoughts all they long, not being in the present moment experience, you could be suffered a lot. Negative thoughts will naturally arise periodically, and you will be suffered from being caught up in those thoughts. If you do not bring your attention back to the present moment experience regularly, you will not be able to notice what you are thinking. You will not realize that you are suffering because of thoughts, and cannot be free from them.

Being aware of the thoughts that you are constantly thinking all day allows you to choose whether you will continue thinking those thoughts or stop thinking about them and focus on something else. Being able to choose to think further or stop thinking is essential for reducing suffering in your life.

Thoughts sometimes are useful and we need to use them such as when we work, when we want to understand or plan something. In this case, continuing thinking further is beneficial. You do not need to stop thinking. You just let thoughts doing their work when you need to do work or think about something. When you do not have to think or work, you can simply keep paying attention to the present moment experience. This will be the middle path that is not too focused or distracted.

After you practice mindfulness for a while, you will become thoughts user (use helpful thoughts to think, plan, or work, and let go of suffering thoughts), not whom be used by thoughts (carried away by the thoughts and suffering from the things that the thoughts create).

Day 18

Desire has many benefits, such as giving us a goal in life, making us want to develop ourselves.

Nevertheless, desire could also make us suffer a lot if we want something that is impossible or very difficult, such as wanting other people to change their habits, wanting to fix the past, wanting everyone to always be satisfied.

Usually, when we have a desire for something, there will be some pressure occur in our minds that motivates us to do something. This pressure in your mind is small hidden suffering. If you want to drink coffee and then get some coffee, this pressure will disappear and you will be satisfied. Therefore, you may not see suffering that come with pressure from desire. However, if you cannot get coffee you want, you may feel annoyed or frustrated. When problematic system in your workplace cannot be corrected or improved as you want, the desire in your mind may cry out and make you feel distressed.

People naturally learn that when they desire something, they have to get what they want first and then their desire will disappear. Indeed, although you cannot get what you want, when you direct your attention back to the present moment experience, your desire can disappear as well.

Desire is like other emotions that have thoughts as fuel. When you have mental images of joyful events, either past event or just your imagination, (such as an image of you eating your

favorite food, an image of you being rich, an image of you having a good time with ex-boy/girlfriends, etc.) desire will begin to form in your mind and urge you to find a way to satisfy it.

After practicing mindfulness for a while, we know that when the thoughts that fuel emotions run out, the feelings are also extinguished. Therefore, when you notice your desiring thoughts arising, you may just bring your attention back to the present moment experience. After a while, your desire at that moment will disappear because your attention is no longer focused on the thoughts that fuel your desire.

Dealing with desire by doing nothing, just bringing your attention back to the present moment, is very useful. You will not have to struggle because of your desire as much as before. Some people who want to lose weight can use this technique when they want to eat some sweets. Some people may use this technique to move on from their ex-boy/girlfriend or deceased loved one. Addicts can also use this technique to help them quit substances as well.

Desire is not something that needs to be eliminated and also cannot be eliminated. Just being able to be aware of your desire, understand the origin of it, and deal with it properly for each situation is enough.

Day 19

Many people may wonder what the endpoint of the mindfulness practice pathway will be. Will they feel no suffering anymore? Indeed, even though you have practiced mindfulness a lot, suffering will still exist. However, it will become lighter in degree and shorter in duration. Suffering or various negative emotions such as irritability, sadness, regret, fear, boredom, discouragement, and frustration are parts of our life as well as positive emotions like happiness. On each day of our life, we will revolve around happiness, suffering and neutral feeling. It will always be like this. Practicing mindfulness will help shorten the time of suffering each time it comes. As a result, our life will be happier.

Deeply in every human mind, there is a desire not to suffer at all. When suffering arises, we try to eliminate it. We may think why it needs to happen to us. The desire to avoid suffering is one of the major causes of suffering. We will not be able to do that and we will suffer more every time suffering comes. When we practice mindfulness, the desire not to suffer at all will be gradually reduced because we will gradually become aware of the truth that there is no life without suffering. Suffering will constantly come into our lives, and it will be like this until the last day of our life. We will be more open to suffering when it occurs in our lives. We will not suffer much when suffering occurs because we understand that it is normal for life to have suffering as its part. We will know that the suffering will come and go. It is only transitory. Just bringing our attention back to the present moment for a while, suffering will fade away. No matter how many times it comes, we can cope with this method. Suffering is not that scary. It is also necessary for our lives because it allows us to grow, develop ourselves, understand the feelings of other human beings, and have compassion and empathy for others.

Rejecting the suffering may cause additional suffering. By accepting them, you will suffer less, and suffering will gradually fade away.

Day 20

We all want happiness. Most of us know and get used to future happiness. However, not many people know about present happiness.

Future happiness is the happiness that you will get when you go through certain happiness conditions. For example, “I will be happy if I have a lot of money”, “I will be happy if I am more beautiful/handsome”, “I will be happy if I can pass the university entrance exams”, etc. If you set easy happiness conditions such as just having rice to eat, you will be happy easily. However, if your happiness condition is difficult or requires a long time such as “I have to be better than everyone in the class”, it will be hard for you to be happy.

For future happiness, when you have not yet passed the condition, your mind will suffer and look forward to future happiness. We may sometimes have to wait for happiness for a short time, and sometimes have to wait for a long time. If happiness condition is not met for a long time, we could be suffered for a long time. If the happiness condition is met, we may be happy, but only for a while. After that, we will return to feel neutral and looking for new future happiness, creating new happiness conditions like this on and on.

Another kind of happiness is present happiness. This kind of happiness is already available in the present moment. You do not have to wait for it. You can experience it by just bringing your attention back to the present moment experience. There will be this kind of happiness available every time you are in the present moment. The examples of present happiness are feeling of relaxation when taking a deep breath, feeling of calm when you are listening to the sound of wind or water, feelings comfort when leaning back in the chair, feeling fresh when drinking some water, etc. You will not experience these kinds of happiness if you are paying attention to thoughts rather than present moment experiences.

There are many kinds of present happiness. Each people may experience differently. You can share with me what present happiness you observe.

Many times, we may be like someone who wears glasses called "happiness" on our heads while trying to look for happiness. Happiness is not anywhere far, but it is here with us all the time. Take a deep breath, and then you will find happiness at the tip of your nose.

Day 21

You may find that on some days you can do well in mindfulness practice, being able to stay in the present moment and sink less in suffering thoughts, while on some days you cannot. This is absolutely normal.

In everyday life, we unintentionally train our attention to be with thoughts, and we do not realize this. We have to think while studying or working for many hours, making our mind think even after working/studying hours. We then continue to think about past or future problems and suffering. If we cannot intermittently bring our attention back to the present-moment experience, we may get lost in thoughts for a long time.

Despite practicing mindfulness for a while, our mindfulness skills can also deteriorate if we pause practicing mindfulness for some time, because we train our attention to be with thoughts every day. To make the effect of mindfulness practice last in the long term, we need to adapt the mindfulness practice to our daily routines, such as showering, brushing teeth, eating coffee, walking to work, etc.

I started with the shower first because I do it twice a day. I felt sensations when I washed my hair and rubbed soap on my body. I listened to the sound of water flowing and hitting the floor, smelled the shampoo, and looked at the shower. When I realized that my mind wandered, I gently returned my attention to the present-moment experience, continued to feel body sensation, and listened to the sound of water.

After practicing this every day, mindfulness practice gradually became a daily routine. I was mindful every time I took a shower. The showering became like a warning bell that reminded me to let go of bad things happening during the day. When I became more accustomed to practice, I gradually expanded my practice to other daily activities. Later, I practiced when I walked to work by continually paying attention to the sensation on my soles, listening to the sounds of cars on the road, and looking at the footpath and buildings. If I realized that my attention was on mental images or mental voices, I gently put those thoughts down and then returned my attention to the present-moment experience. Hence, walking to work gradually became a mindful time in everyday life.

If you can incorporate your mindfulness practice into daily activities like this, your mindfulness skills will be difficult to deteriorate because your mind gradually becomes more familiar with being in the present moment rather than being with thoughts. When you are in the present moment more often, you will recognize that your life is much more comfortable than before. There are rarely things that can disturb you or cause you to suffer much.

You can try to look for activities that are suitable for mindfulness practice and make them mindful moments in your daily life.

Day 22

Being kind to yourself is also important for helping you suffer less and be happy more easily. Being kind means forgiving yourself when you make mistakes, supporting yourself, being okay with yourself, satisfying who you are, etc.

The simple spells that I usually use are the words "it is fine" and "it is okay".’ For example, “it is ok to be rest, I am tired now, I do not have to rush to finish the work that much”, "I am just ordinary people. I have feelings. It is okay to be sad or crying. Finally, it will be passed.” "It is okay, it is normal for making some mistakes, I will try to make amends later." "It is fine to be this far. It is good enough." "It is totally fine to think about it again. It is normal."

The one who stays with you the most in life is yourself. If you can be kind to yourself, understand yourself, support yourself, and accept yourself unconditionally, your life will suffer less. When you suffer, it heals quickly.

Practicing mindfulness may help you recognize the mental voices that judge or blame yourself when you are lazy, make a mistake, or work is unsatisfied. These thoughts may help you improve yourself, but you do not need to listen to them repeatedly. When these mental voices repeatedly talk, you may just gently bring your attention back to the present-moment experience.

Practicing mindfulness does not make one feel happy or sad with anything. You will still be a normal human being who feels. You will still be sad, irritated, afraid, happy, or cry. However, you will be able to understand and accept yourself. You know that every feeling will come and go. You will not sink into suffering as much as before, and deal with suffering better.

Day 23

Our thoughts are similar to movies that project our minds. This comes with both pictures and sounds. The images and sounds of thoughts are constantly changing, scene by scene, and like a movie. Some of these are scenes of a story that has already occurred. Some scenes are imaginations that have not yet occurred.

When you are really into movies, you may have some emotions depending on what scene in the movie that you watch. You may be angry when the villain bullies the leading actress. You may cry when the leading actor and the actress are separated. You may smile and be happy when the leading actor and actress are satisfied with love. The more we enter that movie, the more we

feel. We may realize for a moment that we are watching a movie, not an actual story. When we are in it, we feel like what is going on in the movies is happening in our lives.

Likewise, thoughts are just thoughts and not facts. When you are really into it, there can be many emotions occurring, and you may feel like what you are thinking is happening right now. You will not realize that these are just thoughts, not facts. For example, think of one of your colleagues. You think that he might not like you, but he does not express his feelings to you. When you do not realize that it is just your thought, not a fact, you may become immersed in these mental images and sounds. Then you may start to get angry with him, how can he feel like this to me? You may start to feel tired when you have to work with him, and may start to worry about how to talk to him to make him not hate you. Nobody knows if he does not like you. However, your mind has already created a movie with pictures and sounds for you to watch. When you cannot differentiate between thought and reality, you will be easily immersed in your thoughts and suffer like when you are in movies.

Another example is that your mind might create a past event scene in which you have a fight with your close friend. His mental voices keep saying that "He does not really care about me. He only thinks of himself.". When you are in this mental scene, you may start feeling angry or sad. Although, in reality, no one knows whether he did care about you on that day, your mental voices keep telling you that he did not. If you are really in this mental movie, this movie probably plays further in your mind for an hour. However, if you realize that it is just a thought and know that it does not mean that all you think is all of the facts, you may become more mindful, be able to see a thing from different perspectives, and be able to return your attention back to the present moment experience to calm down your anger or regret.

Thoughts are just thoughts and not reality that is happening right now. A thought is merely a mental image or perspective. When you cling to and so believe in your thoughts, you can easily suffer. Our thoughts tend to be biased by nature. Thoughts often judge things as good or bad, and do not realize that things have both good and bad parts. Some people tend to blame themselves as the cause of mistakes instead of multiple factors. Some people often think that bad things will happen in the future, despite the fact that good things can also happen.

If you can reduce your tendency to overly cling to or believing in your thoughts, you will be able to eliminate one significant cause of suffering.

Day 24

Many people might have times that they do not want to work. This is what most people feel. If you have this experience, you have many friends who feel the same.

Laziness may have several advantages. It can drive you to find a way to finish work as quickly as possible. It could help you unintentionally develop skills to work quickly. However, it could be an obstacle for your work and make you unhappy many times.

Some people are stuck in a cycle called "lazy – blame yourself". They do not want to work, so they do not work. Then, they feel bad for themselves and blame themselves for being lazy and irresponsible. When they have to work, they do not feel like they are working or suffering. If they have to do the job they really dislike, they suffer even more. Understanding the nature of this laziness may help you live more easily and happier.

After practicing mindfulness for a while, you will see that laziness comes into mind occasionally. You may notice that laziness also has thoughts as a fuel, similar to other feelings. When you think of work that you have to do but do not want to do, a feeling of laziness will arise immediately. However, when you bring your attention back to the present-moment experience,

laziness disappears. When you have been working for a while and are very focused on that job, laziness disappears as well. Sometimes, you could be so focused on your work and forget to feel lazy. When you do not focus further on your work and start thinking about the remaining work or time to take a break, the feeling of lazy will come back again.

The key point is that trying to focus on your work as much as possible will give you less time to have lazy thoughts. You may take notes during a meeting or make a summary, diagram, or PowerPoint while reading. It may be difficult to oppose laziness at the beginning. However, if you remain in the present moment, not being with thoughts, it will be less difficult. After focusing on your work for a while, you will not feel tormented by laziness again. Just start doing what you have to do; do not have to care for your laziness. You do not have to wait for laziness to go away or feel like you are doing your work. Just do it, and you will forget your laziness.

For those who cannot concentrate on something for a long time, you may divide your work into small parts that can be done in less than half an hour. For example, you may set your goal to finish a chapter of a book or complete half of your presentation within half an hour and rest after that. Then, you will continue to work again after taking a break. When the work is subdivided, it is not difficult to finish. You hope that your work will finish and be less lazy. Conversely, when you think of work that takes weeks to complete, you feel lazier. Do not forget to reward oneself after completing each subtask. You may eat snacks, watch TV, play on your mobile phone, or whatever.

When you understand the nature of laziness, you can get stuck less in the lazy-blame cycle.

Day 25

Is mindfulness practice the same as distraction practice? Mindfulness practice has you direct your attention away from negative feelings or suffering thoughts to the present moment experiences (body sensation, surrounding sounds, and front images). When your attention is no longer with negative thoughts or feelings, your suffering disappears. This part is like a distraction technique in which you find something to do (like watching movies, getting something to eat) when you suffer from distracting oneself from negative thoughts and feeling better.

However, mindfulness practice has many aspects that differ from distraction techniques.

1. Mindfulness practice does not require movies to watch or eat food. You can use what is already with you at any time, such as body sensations, surrounding sounds, and front images, as something you can focus your attention on. Therefore, you can bring your attention back to the present-moment experience and end your suffering whenever, wherever, and as often as you want.

2. Paying attention to the present-moment experience can cause one to pause and think. Just look, listen, and feel your body. Do not have to judge whether it is good or bad, like, or dislike. Just be aware of the images, sounds, and body sensations. In contrast, distracting oneself by doing other activities simply changes the topic of what you are thinking, not pausing from thinking, which can make you get stuck in other negative thoughts.

3. Regarding attitudes when practicing mindfulness, we do not object to negative thoughts or feelings. We know that it is the nature of thoughts and feelings to come and go. Therefore, we cannot prevent them from arising. However, they will not last long when they arrive. When we return our attention to the present-moment experience, they disappear. We perceive these as images and sounds that come and go at each moment. Just be aware of them— there is no need to interpret them as good or bad, like, or dislike. When you are neutral, not trying to get rid of suffering or frustration, you will suffer less. On the other hand, the aim of distracting yourself by doing other activities is to eliminate suffering. If you try not to suffer, when suffering comes, you

will suffer again. You may be frustrated and ask why it has to happen. Because we have not yet truly understood the nature of suffering that cannot be prevented, we suffer when suffering comes.

Day 26

Have you ever felt drained or fatigued after joining a long conference or lecture? Not only your body that is tired, but also your mind. This can arouse many negative emotions. Some people may feel annoyed and think "You talk too much." "Why are you arguing, what for?" "That person will do like that. This person will do so. Sigh!" "I don't see the point at all" “you never listen to me when I speak” "when I asked did you like it, you said nothing, but then you gossiped with others behind my back.” "Does this matter need a meeting?" "Does the room temperature have to be this cold?" etc. Some people may feel bored and think, ‘When will it end?’ "I want to eat ice-cream" "Aww, my back is so painful". These mental voices may keep talking and cause you to become more exhausted.

If a meeting or lecture is frustrating and boring, you can use this opportunity to practice mindfulness as well. Take a long deep breath, stay aware of the feeling of your breath, feel the sensation of your feet touching the floor, and feel the sensation of your back leaning against the chair. If any thought arises, watch it and let it go like when you let each sound around you pass by. Take a deep breath. Keep listening to sounds. It is sometimes an outer sound and sometimes an inner voice. Just watch them and let each sound come and go. There is no need to interpret, try to understand, or give meaning as good or bad. You may be fully concentrated on the meeting only when you have something that requires focusing.

For example, one may hear people talking, and then the sound of an air conditioner. Then, your inner voices say, ‘Why is the room temperature so cold?’. After that, you hear the sound of the chair moving, and return to hear the people talking again. Then, you hear your mental voice say, ‘When will it end?’ and hear the voices of the person next to you talking with his friend...

Keep listening to this. Let each sound or voice pass through it. Take a deep breath. It can calm your mind and recharge your energy. You also have more opportunities to practice mindfulness. It will be easier for you to stay in long-boring meetings or lectures. It is a way to deal with the boredom. You may use this technique when you have to wait for somebody or something.

Day 27

I have some tips for reducing suffering to share with you. You can apply it in many situations, regardless of what your problem is or how much suffering you feel.

1. If the problem can be solved, but you do not know how at the moment, you may take a break and bring your attention back to the present-moment experience. This may take hours or days. If this can be solved, you will eventually figure it out. Our brains can solve problems better when they are peaceful or neutral. If you are stressed, it would be more difficult to solve this problem. The thinking brain will not function properly when you are too stressed. Outsiders tend to see problems thoroughly and are able to solve problems better than problem owners. This is because they are not drowned in problems or solve problems with a neutral mind. Being mindful will allow you to solve problems better like outsiders.

2. Some problems cannot be solved, for example, wanting to correct the past, wanting to not think about something, wanting not to feel something, etc. The more you think about it, the more stressed you feel because it is impossible. If the problem cannot be solved, stop further thinking and then gently return your attention to the present moment experience. Further thinking only causes you to suffer.

3. The suffering at that moment will eventually pass like every other suffering in the past. How much do you suffer? How much do you deal with your challenges in life? If you understand the nature of suffering, that it will pass and often bring your attention back to the present-moment experience, you will suffer less and suffering will gradually go away.

4. What other people say about you is simply a thought. The thought is merely a perspective, not all of the facts. Therefore, what others say about you is not the entire you. You could suffer so much if you completely believe that what they say about you is all of you.

5. Our thoughts are based on our experiences. Other people's thoughts come from their own experiences. No particular thought is more correct or better than another. These are different perspectives. You can suffer so much if you believe in any thought and think that it is the best and only truth.

6. Happiness can be observed at every moment. Many times, we cannot see this because our attention is focused on suffering in the past or happiness in the future. Having rice to eat can lead to happiness. Sitting, lying down, and sleeping can lead to happiness. You can take deep breaths and be happy. Look around and see what you have at that moment. You can be happy if you see and are satisfied with what you have, such as good health, being capable of taking care of yourself, having a job and a family, having money, having food to eat, etc.

I want to encourage you to apply mindfulness practice to every activity of your daily life. No matter what you are doing, just listen to the sounds around you, look at the image in front of you, feel the body sensation, and be aware of the thoughts and feelings that arise. When you get used to it and become a habit, your mindfulness skills will remain with you in the long term.

Day 28

You can assess the progress of mindfulness practice by yourself. You may assess it by looking at how often you can be aware of your thoughts in a day, how often you pay attention to the present moment experience, and how often and how quickly you can let go when you suffer. You will be the person who knows the best about your progression.

Burnout, stress, depression, and anxiety have the same root cause: being caught up in negative thoughts. Mindfulness helps you to be aware of your thoughts and step back to being the observer who knows that they are just thoughts, not facts. It helps you not be overwhelmed by these thoughts and chooses whether to continue thinking. Therefore, mindfulness is like a remedy that helps alleviate these conditions and helps you have a better quality of life because you can see happiness in the present moment more clearly.
