# Supplementary material for: Efficacy and Feasibility of the Minimal Therapist-Guided Four-Week Online Audio-Based Mindfulness Program ‘Mindful Senses’ for Burnout and Stress Reduction in Medical Personnel: A Randomized Controlled Trial
Source: Healthcare (Basel). 2022 Dec 14;10(12):2532. doi: 10.3390/healthcare10122532 (PMC9778772; doi:10.3390/healthcare10122532)
Supplement: Supplementary file 1 [file healthcare-10-02532-s001.zip › PSA contents S1.pdf]

Appendix SB. Contents of the four online Psychological Self-Help Articles (PSA)

1st PSA - Burnout Syndrome

What is burnout syndrome?

Burnout syndrome is not a disease or a sickness. It is a condition caused by chronic stress from work. Symptoms include feeling powerless, exhausted, having negative feelings about work, feeling empty, or not being able to work as well as before. Nevertheless, if these symptoms are not caused by work, they are not called burnout syndrome (according to the definition of ICD-11).

What are the signs of burnout syndrome?

1. Behavior - You may become critical of your coworkers or clients, get angry easily, eat more, or use a substance to treat yourself.

2. Feelings - You may not be willing to do anything, or you might be lazy to work. After you finish your job, you are not satisfied.

3. Physical change - It might be hard to sleep, or you might be able to but sleep more than you usually do. You might also have headaches, abdominal discomfort, or even irritable bowel syndrome.

4. Productivity - You may find it hard to focus on your work.

These are just signs to look at to see if you have burnout syndrome or not. You may not have them all.

What causes burnout syndrome?

Burnout is caused by two main factors: environments and ourselves.

1. Environments

1.1. Workload – The kind of work that easily causes burnout syndrome is the one which has a great deal of work that is too much for a person to do. It cannot be done in the given timeframe or it has to be fixed over and over again, which makes the total amount of work too much. Burnout syndrome can happen if you work too long hours, have no set work hours, or have to bring work with you to your home when you should be able to rest. When you have to live with these things for a long time, your enthusiasm will melt away.

1.2. Working environment - The characteristic of the workplace that can easily cause burnout syndrome is lacking of freedom in decision-making. The workers must follow as the superintendent commands only. They always have to ask their boss before doing anything. They cannot think on their own. If they ask or disagree, they will be viewed as stubborn person. They cannot say anything, even express their feelings, think, change how they work, or even manage their working time.

2. Ourselves

Some of our habits can make it easier to become burned out:

- Devoted helper - The people who have this trait will think it's perfectly fine if they get tired because they did something nice for someone else. They will think it is a good thing to do. This virtue is indeed fantastic. However, if they don't let themselves get some rest, burnout syndrome will happen sooner or later when their body and mind are too exhausted.

- Perfectionism - People who have this personality type have high hopes for things. They want the job to be done perfectly right. This will take more energy and more time than other

people in order to finish the task. When there are lots of works to do, they cannot finish their jobs in time because it is unacceptable for them to let the work imperfect. Finally, they will be burnout from work overload.

- High responsibility - People who have this tendency will not be able to say no when someone asks for help. Some even take many jobs because they do not want to make other people unhappy or dislike them if they didn't. They receive unlimited jobs without considering their capacity to finish all of the work. Then, the amount of work they had to do in one day was too much for them to do. Finally, they become burned out.

Who is at risk of having burnout syndrome?

People at risk of burnout syndrome include doctors and nurses, housewives, caregivers of the elderly who can't help themselves, caregivers of dementia patients, caregivers of bedridden, caregivers of chronic or terminally ill patients.

How is burnout syndrome treated?

Burnout can be treated by taking a break. People who are burned out must know how to take a break.

1. Take a break from work, set aside some time for rest and not do any work during that time or go on a trip.
2. Get enough food, but do not eat too much, and do not starve yourself or lose weight during this time because it could make you more angry and tired.
3. Get enough sleep. The body will not be able to rest if you do not get enough sleep, so do not use your sleeping time to do other things like watch movies or play games.
4. Exercise to keep your body healthy and your mind refreshed.
5. Take care of yourself. Do something that makes you happy or calms you down.

How can we prevent burnout syndrome?

1. Proper time management - We should prioritize things in our life such as family, work, health, friends, etc. Then, we figure out how much time to work and how much time to do other things in life. Do not spend too much time on work that could harm other parts of your life and no time for rest. For example, you may work only at the workplace and make your home a place for family and health.
2. Self-improvement - We should work on our own working efficiency, so that we can get better work with less time spent, fewer mistakes, and more concentration on the work at hand.
3. Let go of perfection - Some people are many times more fatigued from their works than others simply because they want the perfection of their works. Training to let go of perfection, lower expectations, and accept some small mistakes will help you be less tired at work and prevent burnout syndrome.
4. Refuse some jobs – Although our work refusal today might make some people angry, but it is for everyone good. We will not suffer from work overload and others will not be negatively affected by the fact that we took the job but did not finish it. In addition, refusing to take some work will show others that we have a limit on how much work we can do. They cannot take advantage of us because they know that we will never turn down work. We can say no politely, but the denial needs to be clear for everyone's sake. However, even if we try to be kind, some people who are rejected may not be happy with us. That is a fact that we need to accept and

lower our expectations to please everybody. To expect everyone to be satisfied and not angry at us is not possible.

5. Set a working timetable - It is best for people who have to rely on things outside of themselves to control their behavior to set a schedule to work. We should determine when do we start, and how long will we work for. Then, when we reach some of our goals, we should reward ourselves with something that will help us keep going, like a cup of tea or a movie.

6. Getting someone to help with work - When you have to look after people who are old or sick, it is a hard job. There are a lot of things to do and very little time for rest, so we should look for someone who can take turns. This will help relieve the stress of the caregivers. The caregivers should be able to take a break so that they do not get burned out. It might be a good idea to set the shifts of caregivers so that they know how long they work and when they take breaks.

2nd PSA - Stress Management

What is stress?

Stress is a state in which we have negative feelings such as worry, anger, regret, etc. Some people generally call these feelings suffering.

Stress is a common condition that we all experience in daily life. Although we do not like the feeling of stress, it has some benefits. For example, anxiety makes us prepare for situations and make plans. Anger lets us know what we want and do not want. Regret lets us know that we need support. However, too much or too long stress can have negative effects on our physical and mental health and it may affect our works and relationships.

How do we know if we are too stressed?

A little stress is something that happens every day of your life and it is useful, as mentioned above. However, if the stress is too much, proper stress management is important, before excessive stress destroys your physical, mental health and relationships.

How to determine that we are too stressed? It can be found out by three stress characteristics:

1. Severity of stress

Stress can show how severe it is by how it affects your work, relationship, or happiness. Stress that causes some distraction while you are working is not severe as the stress that stops you from working. Stress that does not affect your relationships is less severe than the stress that makes you fight or curse with your friends and family. Stress that makes us unhappy and feel like the happiness in life is gone is worse than the temporary stress that we still be able to enjoy our life despite the presence of stress.

2. Duration of stress

We sometimes keep thinking about something stressful for a long time. The longer we keep thinking, the more it indicates how stressed we are. For example, a woman said badly to a friend. Then, she felt guilty for what she said for a few hours. After that, she realized that people could make mistakes, so she said sorry to a friend. At last, she did not feel guilt and keep thinking about what she said to a friend anymore. The woman in this case would be less stressed than a man who cannot forgive himself and keep blaming himself for some mistakes for months.

3. The frequency of stress

How often we think about a particular stressful event can also indicate how severe the stress is. Worrying that work will not be finished on time for 20 times per day indicates that a person is more stressed than thinking about it once or twice a day.

We may be able to roughly tell how stress we are by looking at these three characteristics of our stress.

What are the signs of stress?

When we are stressed, we do not just feel bad, but we also have some physical reactions to the stress. When we feel angry, we may breathe faster, feel tightness and uncomfortable in our chest. When we are worried, we may have headaches, faster heartbeat, sweating in our hands, rapid breathing, or stop breathing. When we regret, we may feel physically exhausted, chest pain, etc. This is because the part of the brain that controls emotions is linked to the part that is involved in the control of various organs in the body. So, when we have emotions, we tend to have physical reactions as well.

How does stress negatively affect us?

Stress can adversely affect us in four main areas: physical health, mental health, work, and relationships.

1. Physical health - If we have stress for a long time, it may exacerbate symptoms such as migraine, tension-type headache, fibromyalgia, gastritis, irritable bowel syndrome, coronary heart disease, high blood pressure, etc.
2. Mental health - If we have chronic stress, it can trigger many psychiatric diseases such as depression, bipolar disorder, schizophrenia, generalized anxiety disorder, obsessive-compulsive disorder, insomnia, etc.
3. Work - Stress can cause us unable to focus on work because we may keep thinking about stressful things, or our minds may not be ready for work. We may feel fatigued easily and be less efficient at work.
4. Relationships - Stress can make people fight more often with their coworkers or close ones. It may make us want to stay alone and not meet anyone. Then, we may lack socialization.

How to cope with stress?

There are three main ways to deal with stress: changing your environment, changing your thoughts, and changing how you act

1. Changing your environment

The environment can be a cause of our stress. We may change our environments to reduce or get rid of our stress. For example, changing the environment by going on a vacation and not seeing coworkers for a few days may relieve our stress. Another example, you may keep all the pictures of your ex-boy/girlfriend who recently broke up out of sight if you feel upset every time you see them. When we change our environment in this way, we might be able to relieve some of our stress.

2. Changing your thoughts

Some of our thoughts or mindsets can lead us to stress, so changing some of our thoughts can help us feel less stressed. For example, in a crowded hallway, your friend did not say hello to you when you said hello to him. You thought it might be because you had done something to hurt your friends. Then, you felt stressed. However, if you thought your friend might not see you or your friend might be thinking of something and did not hear you, you might not feel stressed at all. Another example, you did good things, but you received some negative criticism. You thought you worked so hard and nice, how could other people still dissatisfied. Thinking like this could make you suffer and feel frustrated in your mind. However, if you think that it is normal that some people will like what you do and some will not. At least, you have done what is useful to many people. Then, you will not be stressed out.

From the above example, it can be seen that our thoughts can make us happy or sad. Even in the same situation, if we think differently, we can feel different. If we look at things from lots of different angles, not just focus with some negative views, the stress will be significantly relieved.

3. Changing how you act

Stress is sometimes caused by our own behaviors. Some changes in our behavior can help us feel better or get rid of our stress. Suppose you not tell anyone about your stress because you think that no one will get it. You stay away from other people, and do not attend any activities like others. No one will understand your feelings as you thought because you did not meet anyone.

However, if you start to trust someone and talk about your feelings to other people, things can change. There will be someone who can understand you, despite not everyone. If you can change your behavior like this, you could feel less stressed from the feeling of loneliness. Another example, you were afraid that your boy/girlfriend was having someone else, so you often called him/her and checked his/her phone which made him/her feel uncomfortable and he/she started to drift away from you over time. Finally, he/she went to find someone else because he/she could not stand your behavior. If you changed your behavior by giving your boy/girlfriend some personal space. You did not cross the line too far. He/she would know that you respected and trusted her. Then, your relationship would get stronger and you would not have to be stressed because of fear that he/she will have someone else.

How can we prevent stress?

Stress is caused by three factors: the environment, the way we think, and how we act. As a result, you may prevent stress by avoiding stressful environments. If the situation cannot be changed, you should try to notice your thoughts, look for other perspectives or change your behavior. These methods can help protect you from stress. Exploring your thoughts and behaviors is a skill that can be practiced. If we observe ourselves more, we will become more skilled and better at dealing with stress. If we do not know what we are thinking, it can be difficult to see other points of view because we will believe in what we have already thought. If we do not see that our behavior is making us stressed, the stress could become chronic and we might not be able to change our stress-induced behavior.

3rd PSA - Relationship Management

Why do we need to know how to deal with relationships?

All of us must interact with other people on a daily basis, whether it is with our spouses, kids, friends, colleagues, bosses, subordinates, or even people in the society. These relationships have a great impact on how happy we are in our lives. Having a good relationship with a true friend can make us very happy, but having a bad relationship with our parents can make us significantly suffered. We sometimes find it very difficult to have a good relationship with someone, yet it is still possible to improve our relationship with him/her. Knowing how to properly deal with our relationships will help us improve our relationship and will make our lives happier as well.

How can we improve our relationships?

No matter who you are having relationship with, one of the key skills that can help you improve your relationships is communication skill. Communication skill is more than just a skill used for having a normal conversation in everyday life. It is a skill of how to communicate our feelings.

To be able to communicate our feelings well, we need to understand ourselves and others. Knowing how you feel and what you want can help you communicate better. Knowing others' feelings and expectations can help us be able to respond appropriately. For example, a man came home in the evening. When he opened the door, his wife complained "why do you come home late?", "what were you doing?", "Do you care about me?". In this example, it is the communication that worsens the relationship between the couple. If this happens frequently, the man may not want to go home and come home late than before because he does not want to see irritated wife. Then, the wife might get angry more often because her husband tries to avoid her. In a situation like this, it could end up in divorce. Actually, in this situation, the wife felt offended because she thought that her husband was not cared that she was waiting for him for dinner. Deeply, she wants her husband to come home early because it means that he still misses and cares about her. However, this inner expectation cannot be communicated to her husband. What the husband heard was only the complaint and blaming. On the other hand, the husband might have a reason to go home late. He still cares about his wife. However, when he was complained by his wife, he may feel bad or angry and yell her back, leading to a fight. If the wife realize that her husband might feel tired from work. He might want to take a break and had dinner, she might calm herself and gave time for her husband to rest and have dinner before asking the reason why he was late. After that, she may talk to him about how she feels and what she wants. Then, a man could understand her and they could work together to find a solutions. For example, the man may call his wife if he will come home late and tell her the reason. Then, she will know that he cares for her and she will not be worried.

How can we feel less suffering when we must live with people that we do not like?

It is normal to have people that you like and do not like in your life. Likewise, there are some people who like you and someone who do not like you. Even though you did not mean to do something he/she did not like, he/she may still not like you. This is because people are different. They grew up in different ways, so think differently, and like different things. Some people may like people who follow their command and do not say anything, while some people may do not like those who have no comments because those people think that people with no comments have no own idea.

When we meet someone that we do not like, we want to stay away from them. However, if we need to contact with them, what should we do? Someone whom we do not like is actually a normal person who has both good and bad sides like ourselves. They do something because they think it is good, and there are many people who think the same as they are. However, in our view, we think that it is bad. For example, a subordinate who likes to work quickly with no planning may not like a boss who has a detailed plan. He might be annoyed by a boss who pays too much attention to things that he does not think are important. Also, the boss may have a hard time with this subordinate because he sees the subordinate work carelessly and has a poor quality of work. In this example, the boss and subordinates just work in different ways. We may dislike people who do things against what we believe to be good. If we learn to accept people's differences, we will be less stressed. Everyone makes the best choice for themselves. We can learn from other people's good parts and adapt ourselves. When we can see the good parts of others and do not see only the part that we do not like, we will feel less suffering to live with them.

I do not want to do, but I cannot say no. what should I do?

Many people have a hard time saying no, particularly rejecting those who are superior or senior to them. The reluctance to say no may be because they fear being disliked or they think that other people might be angry if they say no. Because of this, you might get a lot of work, feel tired, feel angry, and cannot stand it. However, you do not dare to return the works you got. In the end, there is a chance that you will get burnout syndrome or be depressed.

Assertiveness is very important for people who cannot say no. Standing up for yourself is not selfish or self-centered. It is about giving the value of your feelings as much as anyone else's. The main problem of those who do not dare to say no is that they put the needs of other people ahead of their own needs. They try to make people satisfied, not angry. They think it is okay, though they are not okay. Eventually, they feel tired and can no longer do all the work they planned to.

To stand up for yourself, you must accept the fact that you cannot always please everyone. You cannot always meet the needs of anyone. Let other people be disappointed sometimes. They will learn that you cannot be taken advantage of by them. There may be sometimes that even if you do your best to please others, they are not satisfied. You will be exhausted if you ignore your feelings and keep running to please others.

If you want to say no when you feel like you cannot do as others wish, the important thing is good communication skills. You can say that you cannot do something. You can say that you are feeling tired. You can tell people what you are willing to help and how much you can help them. You may also think about how others feel at the same time. You may need to figure out your own words that would probably make the other person feel okay and you still insist on refusing. You need to know your limits. Doing this will help your long-term relationship with others because no one has to keep their suffering inside, and both can talk about how they feel.

4th PSA - Mental Health Promotion

What is good mental health?

People with good mental health can handle well with any problems that come into their lives. No matter how big or small it is, they can handle it and get through it. People who have good mental health are able to know themselves, understand others, look at things from various perspectives, and let go.

How can we have better mental health?

Observing yourself as much as possible may be the first step. How do you act, think, and feel in different situations? Try to learn from other people about how they express themselves, what they do in the same situation, what they think and feel, and how they deal with the same thing. The more you look at people, the more you will be able to understand them better. You may use different opinions to teach yourself a broader view of the world. If you are more open to different ideas, you might be less miserable or happier. Let me give you an example.

Some people may feel very distressed because they always compare themselves to other people. Why do I not have money like him? Why do I not be as good-looking as her? Why do I not be smart like him? Why do I not have many friends like her? When it comes to that, some people think differently. They think they are so lucky that they have enough money to eat good food, and are healthy enough to go anywhere by themselves. Some people want to do it like this, but they cannot. They are so lucky to have a soft mattress that they can sleep on. Some people do not even own a house. The people in the latter example will be happy with their lives and grateful for everyday because they see the world in different ways.

From the example, you may be able to see that the comparison is only your own idea of what is going on. When you look at the positive side of having something that other people do not have, you will not feel bad as if you looked at the negative side. If you can look at things from many angles like this, it will improve your mental health. You will not be as easily distressed as you used to be because you already knew that you also had something that many others did not have.

Another way to improve your mental health is to change your behavior. Some changes in your behavior can help you learn new things and see things in a new way. For example, a person does not trust anyone because he/she used to be betrayed by a close friend in the past. He/She believes that no one in this world could be sincere or trustworthy. He/She chooses to eat alone and does not go anywhere with coworkers. Although he/she has no risk of being betrayed, he/she is not really happy. He/She still suffered because he/she constantly thinks about his/her friend's betrayal. If this person changes his/her behavior, trying to be more open and trust his/her new coworkers, he/she may be able to make friends who are close and trustworthy. Consequently, it could change his/her old belief that everyone in the world is unreliable. He/She probably will forget the suffering in the past because now he/she is happy and has someone he/she can trust. When his/her mindset changes, his/her mental health improves as well because he/she is able to let go of the past and move on.

As the example, we can see that some of our actions are linked to the persistence of our stress. If we do not try something different or do anything that contradicts our own beliefs, we might not learn the new perspectives. Getting out of the safe zone may help us see things in a new way and to suffer less.
