# Supplementary material for: Efficacy and Feasibility of the Minimal Therapist-Guided Four-Week Online Audio-Based Mindfulness Program ‘Mindful Senses’ for Burnout and Stress Reduction in Medical Personnel: A Randomized Controlled Trial
Source: Healthcare (Basel). 2022 Dec 14;10(12):2532. doi: 10.3390/healthcare10122532 (PMC9778772; doi:10.3390/healthcare10122532)
Supplement: Supplementary file 1 [file healthcare-10-02532-s001.zip › Table S1.pdf]

Table S1. Correlation between audio listening statistics and outcome score changes of Group A

Group A (n = 45)															
Outcome score changes from week 0-4[a]															
Audio listening from week 0-4	CBI total	CBI personal	CBI work	CBI colleague	ST-5	HAD anxiety	HAD depression	PHLMS total	PHLMS awareness	PHLMS acceptance	QOL total	QOL physical	QOL psychological	QOL social	QOL environment
Total listening, minutes	-0.018	-0.094	0.019	0.047	-0.197	0.084	0.091	-0.269	-0.147	-0.245	-0.095	0.115	-0.259	-0.013	-0.140
Total listening, times	-0.017	-0.082	0.020	0.040	-0.201	0.044	0.074	-0.265	-0.103	-0.276	-0.068	0.108	-0.208	-0.008	-0.122
≥3 times a day, days	-0.022	-0.077	0.060	-0.013	-0.181	0.010	0.009	-0.200	-0.027	-0.250	-0.082	0.045	-0.262	0.039	-0.074
Outcome score changes from week 0-8[b]															
Audio listening from week 0-8	CBI total	CBI personal	CBI work	CBI colleague	ST-5	HAD anxiety	HAD depression	PHLMS total	PHLMS awareness	PHLMS acceptance	QOL total	QOL physical	QOL psychological	QOL social	QOL environment
Total listening, minutes	0.118	0.103	0.051	0.158	-0.046	0.055	0.035	-0.335*	-0.091	-0.408**	-0.085	0.052	-0.236	0.100	-0.112
Total listening, times	0.114	0.106	0.053	0.149	-0.069	0.037	0.012	-0.312*	-0.050	-0.419**	-0.073	0.037	-0.198	0.095	-0.099
≥3 times a day, days	0.101	0.054	0.037	0.162	-0.052	0.058	0.034	-0.277	0.009	-0.431**	-0.071	0.029	-0.236	0.095	-0.073

Abbreviations: CBI, Thai version of the Copenhagen Burnout Inventory; ST-5, The Stress Test Questionnaire; HAD-anxiety, The Thai version of HADS anxiety subscale; HAD-depression, The Thai version of HADS depression subscale; PHLMS, The Thai version of Philadelphia Mindfulness Scale; QOL, The Thai abbreviated version of World Health Organization quality of life (WHOQOL-BREF-THAI).

[a] calculated by (mean outcome score at week 4 – mean outcome score at week 0)

[b] calculated by (mean outcome score at week 8 – mean outcome score at week 0)

* $p < 0.05$, ** $p < 0.01$, *** $p < 0.001$ (analyzed by Pearson’s correlation)
