# Supplementary material for: Efficacy and Feasibility of the Minimal Therapist-Guided Four-Week Online Audio-Based Mindfulness Program ‘Mindful Senses’ for Burnout and Stress Reduction in Medical Personnel: A Randomized Controlled Trial
Source: Healthcare (Basel). 2022 Dec 14;10(12):2532. doi: 10.3390/healthcare10122532 (PMC9778772; doi:10.3390/healthcare10122532)
Supplement: Supplementary file 1 [file healthcare-10-02532-s001.zip › Table S2.pdf]

Table S2. Correlation between audio listening statistics and outcome score changes of Group B

Group B (n = 45)															
Outcome score change from week 8-12[a]															
Audio listening from week 8-12	CBI total	CBI personal	CBI work	CBI colleague	ST-5	HAD anxiety	HAD depression	PHLMS total	PHLMS awareness	PHLMS acceptance	QOL total	QOL physical	QOL psychological	QOL social	QOL environment
Total listening, minutes	-0.088	-0.143	-0.187	0.158	0.225	0.076	-0.042	0.178	0.089	0.176	0.066	0.101	-0.097	0.104	0.087
Total listening, times	-0.058	-0.115	-0.166	0.180	0.243	0.108	-0.004	0.205	0.105	0.198	0.032	0.076	-0.128	0.073	0.057
≥3 times a day, days	-0.012	-0.130	-0.086	0.223	0.245	0.026	-0.045	0.129	0.070	0.075	0.013	0.073	-0.118	-0.025	0.040
Outcome score change from week 8-16[b]															
Audio listening from week 8-16	CBI total	CBI personal	CBI work	CBI colleague	ST-5	HAD anxiety	HAD depression	PHLMS total	PHLMS awareness	PHLMS acceptance	QOL total	QOL physical	QOL psychological	QOL social	QOL environment
Total listening, minutes	-0.285	-0.287	-0.210	-0.134	-0.177	0.073	-0.255	0.410**	0.187	0.407**	0.313*	0.306*	0.147	0.333*	0.290
Total listening, times	-0.251	-0.251	-0.184	-0.101	-0.172	0.125	-0.219	0.381**	0.169	0.396**	0.283	0.274	0.118	0.297*	0.256
≥3 times a day, days	-0.231	-0.261	-0.100	-0.067	-0.150	0.120	-0.287	0.321*	0.190	0.264	0.134	0.131	0.097	0.143	0.107

Abbreviations: CBI, Thai version of the Copenhagen Burnout Inventory; ST-5, The Stress Test Questionnaire; HAD-anxiety, The Thai version of HADS anxiety subscale; HAD-depression, The Thai version of HADS depression subscale; PHLMS, The Thai version of Philadelphia Mindfulness Scale; QOL, The Thai abbreviated version of World Health Organization quality of life (WHOQOL-BREF-THAI).

[a] calculated by (mean outcome score at week 12 – mean outcome score at week 8)

[b] calculated by (mean outcome score at week 16 – mean outcome score at week 8)

* $p < 0.05$, ** $p < 0.01$, *** $p < 0.001$ (analyzed by Spearman's correlation)
