# Supplementary material for: Efficacy and Feasibility of the Minimal Therapist-Guided Four-Week Online Audio-Based Mindfulness Program ‘Mindful Senses’ for Burnout and Stress Reduction in Medical Personnel: A Randomized Controlled Trial
Source: Healthcare (Basel). 2022 Dec 14;10(12):2532. doi: 10.3390/healthcare10122532 (PMC9778772; doi:10.3390/healthcare10122532)
Supplement: Supplementary file 1 [file healthcare-10-02532-s001.zip › Table S3.pdf]

Table S3. Correlation between audio listening statistics and outcome score changes of both groups

Group A + B (n = 90)															
Outcome score change from pre-MS to post-MS[a]															
Audio listening from pre-MS to post-MS	CBI total	CBI personal	CBI work	CBI colleague	ST-5	HAD anxiety	HAD depression	PHLMS total	PHLMS awareness	PHLMS acceptance	QOL total	QOL physical	QOL psychological	QOL social	QOL environment
Total listening, minutes	-0.151	-0.257*	0.064	0.062	-0.051	-0.077	-0.095	0.088	0.072	0.049	0.063	0.218*	-0.090	0.090	0.028
Total listening, times	-0.149	-0.253*	0.078	0.069	-0.064	-0.076	-0.092	0.117	0.101	0.065	0.065	0.210*	-0.076	0.082	0.021
≥3 times a day, days	-0.121	-0.233*	0.120	0.086	-0.057	-0.113	-0.102	0.088	0.078	0.027	0.042	0.184	-0.102	0.041	0.006
Outcome score change from pre-MS to one-month post-MS[b]															
Audio listening from pre-MS to one-month post-MS	CBI total	CBI personal	CBI work	CBI colleague	ST-5	HAD anxiety	HAD depression	PHLMS total	PHLMS awareness	PHLMS acceptance	QOL total	QOL physical	QOL psychological	QOL social	QOL environment
Total listening, minutes	-0.125	-0.178	0.093	-0.032	-0.146	-0.107	-0.160	0.110	0.079	0.079	0.185	0.264*	0.049	0.232*	0.129
Total listening, times	-0.133	-0.186	0.101	-0.027	-0.173	-0.106	-0.168	0.123	0.096	0.090	0.191	0.266*	0.059	0.232*	0.134
≥3 times a day, days	-0.114	-0.163	0.151	-0.017	-0.133	-0.079	-0.151	0.062	0.088	0.005	0.098	0.194	-0.002	0.146	0.042

Abbreviations: CBI, Thai version of the Copenhagen Burnout Inventory; ST-5, The Stress Test Questionnaire; HAD-anxiety, The Thai version of HADS anxiety subscale; HAD-depression, The Thai version of HADS depression subscale; PHLMS, The Thai version of Philadelphia Mindfulness Scale; QOL, The Thai abbreviated version of World Health Organization quality of life (WHOQOL-BREF-THAI).

[a] calculated by (mean outcome score at immediate post-MS – mean outcome score at pre-MS)

[b] calculated by (mean outcome score at one-month post-MS – mean outcome score at pre-MS)

* $p < 0.05$, ** $p < 0.01$, *** $p < 0.001$ (analyzed by Spearman's correlation)
